# Soluble E-Cadherin: An Early Marker of Severity in Acute Pancreatitis

**DOI:** 10.1155/2009/397375

**Published:** 2009-04-27

**Authors:** A. Sewpaul, J. J. French, T. K. Khoo, M. Kernohan, J. A. Kirby, R. M. Charnley

**Affiliations:** ^1^HPB Surgical Unit, Freeman Hospital, Newcastle upon Tyne, NE7 7DN, UK; ^2^Department of Surgery, The Medical School, University of Newcastle upon Tyne, Newcastle upon Tyne NE2 4HH, UK

## Abstract

*Background/Aims*. At present, there is no simple test for predicting severity in acute pancreatitis. We investigated the use of an assay of soluble E-cadherin (sE-cadherin). *Methods*. Concentrations of sE-cadherin, from 19 patients with mild acute pancreatitis, 7 patients with severe acute pancreatitis, 11 patients with other acute gastrointestinal pathologies, and 12 healthy subjects were measured using a commercially available sandwich ELISA kit based on two monoclonal antibodies specific to the extracellular fragment of human E-cadherin. Measurements were made at 12 hours or less from onset of pain and also at 24 and 48 hours after onset of pain. *Results*. Mean (standard deviation) concentration of sE-cadherin in patients with severe acute pancreatitis at <12 hours was 17780 ng/mL (7853), significantly higher than that of healthy volunteers 5180 ng/mL (1350), *P* = .0039, patients with other gastrointestinal pathologies 7358 ng/mL (6655), *P* = .0073, and also significantly higher than that of patients with mild pancreatitis, 7332 ng/mL (2843), *P* = .0019. *Discussion*. Serum sE-cadherin could be an early (within 12 hours) objective marker of severity in acute pancreatitis. This molecule warrants further investigation in the form of a large multicentre trial.

## 1. Introduction

More than a century after its comprehensive description by Fitz [1], acute pancreatitis remains a common disorder with potentially devastating consequences. The incidence has been reported to be as high as 38 per 100 000 population per year [2] with around 25% of patients developing severe or life-threatening complications that require high-dependency nursing or intensive care support. Even though the overall mortality over the past 30 years has fallen from 25%–30% to 6%–10%, it has remained at that level for a decade or more [3]. If complications develop, however, mortality increases to 35% or higher [4], with 50% of deaths occurring within one week of the attack, mostly from multiorgan dysfunction syndrome (MODS) [5].

It is generally accepted that all patients with signs of moderate-to-severe acute pancreatitis should be admitted to an intensive care unit and referred to specialised centres for maximum supportive care [6–8]. As complications may develop at any time, frequent reassessment and continuous monitoring are necessary. The most important supportive therapy is adequate and prompt fluid resuscitation with intravenous fluids and supplemental oxygen, with a liberal indication for assisted or controlled ventilation to guarantee optimal oxygen transport [7–9].

To date, inhibition of any known pathogenic step (by, e.g., octreotide, gabexate mesilate, and lexipafant) has not effectively reduced mortality or increased long-term survival in severe acute pancreatitis [10–12]. Thus, treatment of acute pancreatitis in the early stages is still symptomatic, with no specific medication being currently available.

UK guidelines [8] recommend that severity stratification is attempted but presently there is no tool that can predict, with a high degree of accuracy, within 48 hours from the onset of pain, which patients will develop severe disease. Initial clinical assessment alone identifies fewer than half the patients with severe acute pancreatitis [13]. Scoring systems incorporating clinical, biochemical, or radiological criteria for severity assessment have been in use for some decades. These include the 11 criteria described by Ranson in 1970 [14], the Glasgow score (eight criteria) [15], and the acute physiology and chronic health evaluation (APACHE II) score (14 criteria) [16]. The sensitivity and specificity of these scoring systems for predicting severe acute pancreatitis range between 55% and 90%, depending on the cutoff number and the timing of scoring usually reaching optimal sensitivity and specificity at 48 hours [17, 18]. The ideal severity stratification system would be based on a simple-to-perform, affordable, single, and quick test that is widely available and has a high sensitivity and specificity very early in the disease process. The use of single predictive markers has been investigated in the past.

Serum amylase and lipase, the standard tests for acute pancreatitis diagnosis, are poor predictors of severity [19]. Novel markers for the early prediction of acute pancreatitis severity include the pancreatic proenzyme trypsinogen-2 and its subunit TAP [20, 21], as well as early inflammatory response markers such as serum IL-6 [22, 23], procalcitonin [24, 25], polymorphonuclear elastase [26], and serum amyloid A [27]. The more established marker C-reactive protein has been shown to be an accurate severity predictor (sensitivity and specificity above 80%) at 48 hours postsymptom onset if a cutoff level of 150 mg/L is used [6]. Unfortunately, for a variety of reasons (poor predictive value before 48 hours, conflicting reports, and difficult laboratory measurement), none of these single tests have made the transition from the research setting into widespread clinical use.

### 1.1. E-Cadherin

**Figure 1:**
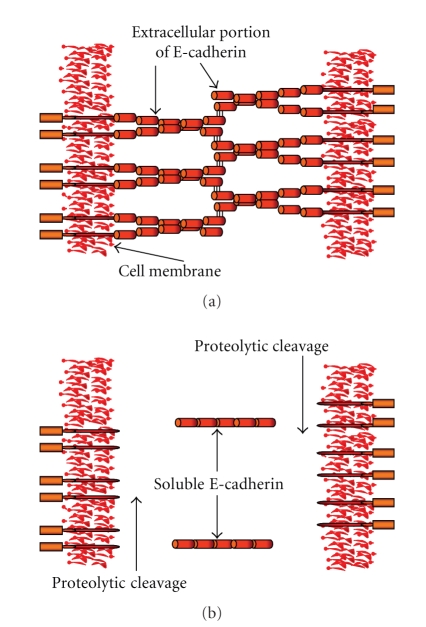
(a) The zipper model for cadherin interaction showing homophilic interactions between E-cadherin molecules expressed on the cell membranes; (b) proteolytic cleavage of the extracellular portion of the E-cadherin fragment generating soluble E-cadherin.

E-cadherin is a 120-kDa transmembrane glycoprotein involved in the calcium dependent adhesion of all epithelial cells. Soluble E-cadherin is an 80-kDa peptide degradation product of the 120-kDa E-cadherin molecule which is generated by a calcium ion dependent proteolytic process [28, 29]. Matrix metalloproteinases, trypsin, kallikrein 7, and plasmin are examples of molecules that are capable of performing this proteolytic process [28–37] (see Figure 1). The maintenance of epithelial membranes in healthy individuals involves a continuous turnover of E-cadherin, and independent of age and sex, low levels of sE-cadherin are found in the serum [38].

Elevated serum levels of sE-cadherin are found in patients with certain malignancies, including pancreatic [30, 36, 38–45]. It has been suggested that these increased levels are due to the cleavage of membrane-bound E-cadherin by tumour-derived proteases.

In a previous study by Pittard et al., significantly elevated levels of sE-cadherin were found in patients exhibiting a systemic inflammatory response. Importantly, these elevated sE-cadherin levels were found within an early time frame of the disease process and correlated to the severity of the developed inflammatory response [46].

A systemic inflammatory response is seen in acute pancreatitis. Therefore, it is probable that sE-cadherin is cleaved from membrane-bound E-cadherin by inflammatory proteases. If this also occurs early in the inflammatory response in acute pancreatitis, and measurable serum levels of sE-cadherin relate to the severity of the developing disease, then this molecule could be used to predict disease severity. To test this hypothesis, we investigated, using a prospective pilot study, whether serum levels of sE-cadherin measured at an early time point following the onset of symptoms (within 12, 24, and 48 hours) in patients with acute pancreatitis were significantly different in patients who went on to develop mild, compared to severe disease. As serum levels of sE-cadherin are elevated in many different inflammatory processes, its use in the diagnosis of acute pancreatitis was not investigated as it is likely to be of limited value.

## 2. Patients and Methods

### 2.1. Study Population

Patients admitted to the Newcastle NHS hospitals with acute pancreatitis were assessed for inclusion in the study. Acute pancreatitis was defined as acute abdominal pain with a typical clinical picture and a serum amylase level at least three times the upper limit of normal and/or typical findings on computed tomography. We excluded patients who had chronic pancreatitis, known malignancies, or were under 18 years. Clinical, pathological, and radiological patient data was collected prospectively on a database.

We enrolled by parallel recruitment a number of patients admitted with other abdominal inflammatory pathologies such as acute diverticulitis, perforated duodenal ulcer, cholangitis, acute appendicitis, and acute cholecystitis. A third group of healthy volunteers was also recruited. The same exclusion criteria applied to the patients with acute pancreatitis.

### 2.2. Study Design

Ethical approval was obtained from the Newcastle and North Tyneside Health Authority, University of Newcastle upon Tyne and University of Northumbria at Newcastle, and Joint Ethics Committee.

Following informed consent, three blood samples, for sE-cadherin measurement, were taken from each individual. The first measurement was taken from the admission blood sample. The subsequent three samples were taken at 12, 24, and 48 hours after the onset of pain. Samples were subjected to centrifugation within 60 minutes and stored at −20°C. sE-cadherin levels were measured using a commercially available sandwich ELISA kit from Takara Shuzo, Japan. In brief, the capture monoclonal antibody is coated onto microtitre plate. Following blocking of nonspecific binding, patient samples, or standard solutions is incubated in the wells at 37°C for 2 hours. The detection monoclonal antibody (conjugated with peroxidase) is then incubated in the above wells at 37°C for 1 hour. The addition of peroxidase substrate solution (H_2_O_2_ and tetramethylbenzibine) results in a colour change. The reaction was terminated by the addition of 1 M H_2_SO_4_. Absorbance was measured with the microtitre plate reader at 450 nm. Each sample was measured three times and a mean value derived. Sample concentrations were determined from standard curves obtained from standard solutions.

Patients with local and/or organ failure were defined as having severe acute pancreatitis according to the Atlanta classification [47].

Data was analysed using GraphPad PRISM (version 3.0). The data for sE-cadherin levels were compared using the Mann-Whitney *U*-test. Differences were considered as significant when *P* < .05.

## 3. Results

### 3.1. Patients

A total of 49 patients were recruited into the study; all met the eligibility criteria. 26 patients had acute pancreatitis, 12 had other acute abdominal pathologies, and 11 were healthy controls.

The overall median age of patients with acute pancreatitis was 56 (range 24–87); 12 were men. 19 patients had mild disease, and the remaining 7 had severe disease. The aetiology was gallstones in 13 patients (9 mild and 4 severe). Alcohol was the cause in 4 (3 mild and 1 severe). ERCP was the cause in 3 patients (2 mild and 1 severe). No obvious cause was found in 6 patients (5 mild and 1 severe). None of the mild cases and 1 of the severe cases died.

The median age of the 12 healthy controls (4 male) was 29 (range 21–54).

The median age of the 11 patients with other acute abdominal pathologies (5 male) was 59 (range 29–72). The final diagnoses for these patients were as follows: gastroenteritis 1; gastritis 1; acute cholecystitis 2; acute appendicitis 2; diverticulitis 1; cholangitis 1; perforated peptic ulcer 3.

### 3.2. Serum sE-Cadherin Levels

**Figure 2:**
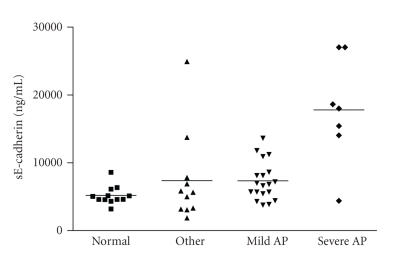
sE-cadherin concentration in patients at 12 hours or less after onset of pain. Data are shown as a scatter plot with the mean represented by a solid line.

At less than 12 hours from onset of pain, the mean (standard deviation) concentration of sE-cadherin in patients with severe acute pancreatitis was 17780 ng/mL (7853), significantly higher than that of healthy volunteers 5180 ng/mL (1350), *P* = .0039 (see Figure 2). In contrast, the mean (SD) concentration of sE-cadherin in patients with mild acute pancreatitis was 7332 ng/mL (2843), and the mean concentration of sE-cadherin in patients with other pathologies was 7358 ng/mL (6655). The mean level in mild acute pancreatitis patients was also significantly higher than that of healthy volunteers (*P* = .0166), but not in those with other pathologies (*P* = .3909). Importantly, significantly higher levels of sE-cadherin were detected in patients with severe acute pancreatitis within 12 hours following onset of pain (mean 17780 ng/mL) compared to mild pancreatitis (mean 7332 ng/mL), *P* = .0019 and those with other gastrointestinal pathologies (mean 7358 ng/mL), *P* = .0073.

**Figure 3:**
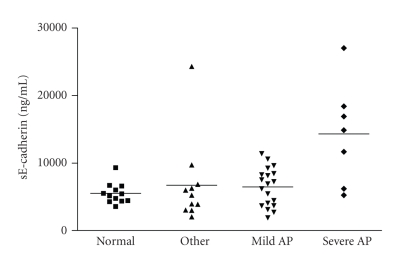
sE-cadherin concentration in patients at 24 hours after onset of pain. Data are shown as a scatter plot with the mean represented by a solid line.

At 24 hours after the onset of pain, the mean (SD) sE-cadherin concentration was 14320 ng/mL (7532) in patients with severe acute pancreatitis, still significantly higher than that of healthy volunteers 5518 ng/mL (1518), *P* = .0030 and also compared to those with mild pancreatitis 6474 ng/mL (2823) *P* = .0065 (see Figure 3).

**Figure 4:**
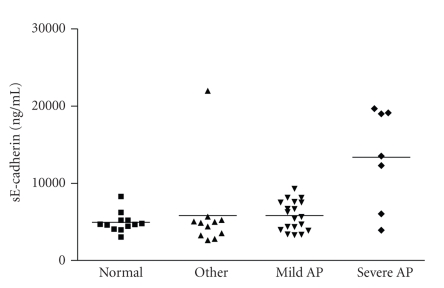
sE-cadherin concentration in patients at 48 hours after onset of pain. Data are shown as a scatter plot with the mean represented by a solid line.

At 48 hours the mean (SD) sE-cadherin concentration was 13360 ng/mL (6440) in patients with severe acute pancreatitis, still significantly higher than that of healthy volunteers 4928 ng/mL (1314) *P* = .0080, and also compared to those with mild pancreatitis 5818 ng/mL (1899) *P* = .0076 (see Figure 4).

## 4. Discussion

In the clinical setting of a patient with acute pancreatitis, initial therapy, accurate severity stratification, and an appropriate facility for patient management are of major interest for the admitting clinician. At present, there is no method by which to accurately predict severity. Many scoring systems have been proposed but all have their drawbacks. The possibility that an affordable, quick, single, and accurate test may exist has led clinicians to investigate numerous (mainly inflammatory mediators) biochemical molecules. Many have been assessed and detected either in serum or urine but for a number of reasons have failed to reach the clinical setting.

This study has shown that serum levels of sE-cadherin can be used to predict severity of acute pancreatitis at an early time point, with the mean differences in sE-cadherin concentration being statistically different within 12 hours or less or at 24 hours after the onset of pain. Furthermore, although not presently widely available, this test is quick, affordable (comparable to CRP), and could easily be incorporated into hospital practice. It is thought that sE-cadherin, elevated levels of which are found in inflammatory conditions, originates via cleavage from membrane-bound E-cadherin. The mechanism by which and at exactly which point during an inflammatory process this occurs is not presently fully understood. Experimentally, molecules such as metalloproteinases, kallikrein 7, plasmin, and trypsin have all been shown to be capable of performing this cleavage process [28–37]. With regard to metalloproteinases, it is known that they can cause organ damage [48] and have a tissue remodelling role in the regeneration following an attack of pancreatitis, but recently evidence is emerging that they could be important in early inflammatory events. Not only have elevated levels of MMP-2 and MMP-9 been reported in the peritoneal fluid of rats following sodium taurocholate-induced pancreatitis [48], but also MMP-3 and MMP-9 enzyme activity has been shown to be elevated 12 hours after cerulein-induced pancreatitis [49]. Therefore, trypsin and/or MMP activation very early in the pathogenesis of acute pancreatitis could be one mechanism by which the elevated levels of sE-cadherin are achieved.

In a recent study, Steinhusen et al. [35] reported that during apoptosis, fragments of E-cadherin with apparent molecular masses of 24, 29, and 84 kDa were generated by two distinct proteolytic activities. In addition to a caspase-3-mediated cleavage releasing the cytoplasmic domain of E-cadherin, a metalloproteinase sheds the extracellular domain from the cell surface during apoptosis. In experimental pancreatitis, it has been found that acute pancreatitis is associated with the induction of acinar cell apoptosis, the degree of which mimics the severity of pancreatitis [50].

The mean sE-cadherin concentrations were higher in our healthy controls compared with those reported in one study (5180 ng/mL versus 2515 ng/mL, resp.) [40], but similar to levels found in a more recent study (5616 ng/mL) [42]. Racial differences between groups have been suggested as an explanation, but we suspect that the ELISA substrate used is the reason. The substrate used was o-Phenylenediamine (OPD) in the former study [40] and 3,3′,5,5′-Tetramethylbenzidine (TMB) in our own and the latter study [42] where comparable sE-cadherin levels were found.

Prior to our study, we suspected that raised sE-cadherin levels would be found in all patients experiencing an inflammatory event. From our results, it appeared that this was not the case, and that raised sE-cadherin levels were only found in patients with an inflammatory insult that resulted in significant multiorgan failure. The solitary high sE-cadherin level measured in the group of patients with other pathologies was from a patient with cholangitis who did go on to develop multiorgan failure.

Of the seven patients who had the severe form of acute pancreatitis, 6 had significantly elevated levels of sE-cadherin within 12 hours from the onset of pain. These patients required admission to critical care for organ support within 24 hours of their presentation to hospital and went on to develop MOF. The remaining patient, whose sE-cadherin level was not significantly raised within 12 hours after the onset of pain, is worthy of further discussion. This female patient presented with gallstone pancreatitis was managed initially on the general ward. A further bout of pain, however, developed on day 4 following admission for which she needed HDU care on day 5. She went on to develop multiorgan failure and pancreatic necrosis. It is possible that there may have been a second attack of pancreatitis induced on day 4 in an inpatient and that she subsequently developed severe acute pancreatitis and necrosis from this second attack. Unfortunately, we do not have serum sE-cadherin levels relating to this second bout of pain.

Despite the small sample size in this study, sE-cadherin seems to be an exciting potential very early marker (within 12 hours of onset of symptoms) of severity in acute pancreatitis. It is noted however that this is a small pilot study and that firm conclusions cannot be drawn until the hypothesis has been tested on a larger population. A larger sample size would permit more sophisticated statistical analysis. The predictive value of sE-cadherin could then be evaluated by receiver-operating characteristic (ROC) curves to determine the optimal cutoff value to predict severe AP. Sensitivity and specificity, as well as positive and negative predictive values, could then be calculated. This would also allow for a useful comparison of the prognostic value of E-cadherin with other predictive scores. We therefore feel that this molecule warrants further study in the form of a multicentre trial.
